# IRF7 expression correlates with HIV latency reversal upon specific blockade of immune activation

**DOI:** 10.3389/fimmu.2022.1001068

**Published:** 2022-09-05

**Authors:** Ifeanyi Jude Ezeonwumelu, Edurne García-Vidal, Eudald Felip, Maria C. Puertas, Bruna Oriol-Tordera, Lucía Gutiérrez-Chamorro, André Gohr, Marta Ruiz-Riol, Marta Massanella, Bonaventura Clotet, Javier Martinez-Picado, Roger Badia, Eva Riveira-Muñoz, Ester Ballana

**Affiliations:** ^1^ IrsiCaixa AIDS Research Institute – IrsiCaixa and Health Research Institute Germans Trias i Pujol (IGTP), Hospital Germans Trias i Pujol, Universitat Autònoma de Barcelona, Badalona, Spain; ^2^ Medical Oncology Department, Catalan Institute of Oncology (ICO)-Badalona, B-ARGO (Badalona Applied Research Group in Oncology) and IGTP (Health Research Institute Germans Trias i Pujol), Universitat Autònoma de Barcelona, Badalona, Spain; ^3^ Consorcio Centro de Investigación Biomédica en Red de Enfermedades Infecciosas (CIBERINFEC), Instituto de Salud Carlos III, Madrid, Spain; ^4^ Scientific Computing Facility, Max Planck Institute of Molecular Cell Biology and Genetics, Dresden, Germany; ^5^ Centre for Health and Social Care Research (CESS), Faculty of Medicine, University of Vic – Central University of Catalonia (UVic – UCC), Vic, Spain; ^6^ Catalan Institution for Research and Advanced Studies (ICREA), Barcelona, Spain

**Keywords:** IRF7, JAK-STAT signalling, HIV-1 cure strategies, latency reversal agents, innate immunity

## Abstract

The persistence of latent HIV reservoirs allows for viral rebound upon antiretroviral therapy interruption, hindering effective HIV-1 cure. Emerging evidence suggests that modulation of innate immune stimulation could impact viral latency and contribute to the clearing of HIV reservoir. Here, the latency reactivation capacity of a subclass of selective JAK2 inhibitors was characterized as a potential novel therapeutic strategy for HIV-1 cure. Notably, JAK2 inhibitors reversed HIV-1 latency in non-clonal lymphoid and myeloid *in vitro* models of HIV-1 latency and also *ex vivo* in CD4+ T cells from ART+ PWH, albeit its function was not dependent on JAK2 expression. Immunophenotypic characterization and whole transcriptomic profiling supported reactivation data, showing common gene expression signatures between latency reactivating agents (LRA; JAK2i fedratinib and PMA) in contrast to other JAK inhibitors, but with significantly fewer affected gene sets in the pathway analysis. In depth evaluation of differentially expressed genes, identified a significant upregulation of IRF7 expression despite the blockade of the JAK-STAT pathway and downregulation of proinflammatory cytokines and chemokines. Moreover, IRF7 expression levels positively correlated with HIV latency reactivation capacity of JAK2 inhibitors and also other common LRAs. Collectively, these results represent a promising step towards HIV eradication by demonstrating the potential of innate immune modulation for reducing the viral reservoir through a novel pathway driven by IRF7.

## Introduction

HIV-1 infection remains incurable due to the persistence of HIV in long-lived reservoirs that support viral rebound upon treatment interruption. Therefore, the current therapeutic option for people with HIV (PWH) is a life-long suppressive antiretroviral therapy (ART), albeit long-term side effects and development of treatment resistance hampering treatment efficacy, and immune dysfunction persists indefinitely (1, 2). One strategy for eliminating latently infected cells is the “shock and kill”, which aims to activate virus transcription, protein expression and virion production using latency reversal agents (LRAs). Then, reactivated cells will be subsequently eliminated by either viral cytopathic effect or host cytolytic effector mechanisms (3). Although some of these LRAs have shown promising potential in reversing HIV-1 latency *in vitro*, reducing the size of the viral reservoirs has remained unsuccessful in clinical trials due to the complexities of mechanisms governing latency reactivation (4, 5). Conversely, the permanent silencing of the latent reservoir and HIV transcription have also been explored in recent years, the so called “block and lock” strategy, which aims to establish a state of deep latency (6).

Latently infected cells can escape the viral immune response and persist for long periods. Latency is primarily established in infected activated CD4+ T cells as they transition to a resting memory state (7). HIV-1 persists in all subsets of memory CD4+T cells, including memory stem cells (T_SCM_), central memory cells (T_CM_), transitional memory cells (T_TM_) and effector memory cells (T_EM_) (8–10). These subsets of memory CD4+ T cells show distinct phenotypes in their transcriptional activity, HIV proviral inducibility, and contribution to the latent reservoir (11–14). Besides resting memory CD4+ T cells, cells of the myeloid lineage, especially macrophages, are believed to be an important sanctuary for HIV-1, due to their ability to spread HIV-1 infection in immune-privileged sites (15). Monocytes and macrophages are key players in the innate immune response to pathogens and are recruited to sites of infection and inflammation. While the establishment of a viral set-point coincides with the downregulation of acute innate immune responses, systemic inflammation persist during chronic HIV infection and, although reduced in PWH on ART, immune activation persists, closely associated with T-cell exhaustion and compromising CD4+ T-cell recovery and immune function (16).

The Janus kinase–signal transducer and activator of transcription (JAK-STAT) pathway is an essential mediator of host immune response to viral infections, including HIV, regulating cytokine secretion and induction of antiviral interferon (IFN) stimulatory genes. Cytokines and IFNs are among the causative orchestrators of chronic inflammation associated with viral reservoir formation and maintenance. Thus, targeting the JAK-STAT pathway has been proposed as an attractive cellular target to achieve HIV suppression and to reduce inflammation concomitantly, due to its specific anti-inflammatory properties in various cell types (17, 18). Indeed, several Janus kinase inhibitors (JAKi) have been developed and approved for the treatment of inflammatory diseases as myelofibrosis and rheumatoid arthritis (19). Recently, JAKi received emergency use approval to treat COVID-19, due to their potent efficacy in reducing type I IFN-driven inflammation *in vivo*, paving the way towards their use as treatment strategies against other viral infections, including HIV-1 (20). JAKi treatment leads to the inhibition of various proinflammatory cytokines, including interleukin-6 (IL-6), tumor necrosis factor alpha (TNF-α), and IL-1 among others (21). In clear contrast to their proposed antiviral role, a commonly reported effect of JAKi treatment in humans is the increased risk of reactivation of chronic viral infections [reviewed in (22)]. Thus, selective JAKi might also induce reactivation of latent HIV while preventing chronic inflammation, representing therefore an interesting new strategy for HIV cure and prompting an in-depth evaluation of their putative role in chronic HIV infection.

Here, we show that a specific subclass of selective JAK2i reverses HIV latency *in vitro* and *ex vivo* in CD4+ T cells from ART-suppressed PWH. Evaluation of the underlying host cellular factors involved, identified interferon regulatory factor 7 (IRF7) as a novel protein putatively relevant for HIV latency reversal, an effect that was independent of JAK-STAT signalling blockade. Overall, these data demonstrate that targeted inhibition of the JAK-STAT pathway may provide a selective, effective, and novel mechanism for purging HIV-1 reservoir in lymphocytes and macrophages, having at the same time a significant impact on HIV-1 induced chronic inflammation.

## Results

### Identification of specific JAK2 inhibitors as latency reversing or latency promoting agents in non-clonal cellular models of HIV-1 latency

**Figure 1:**
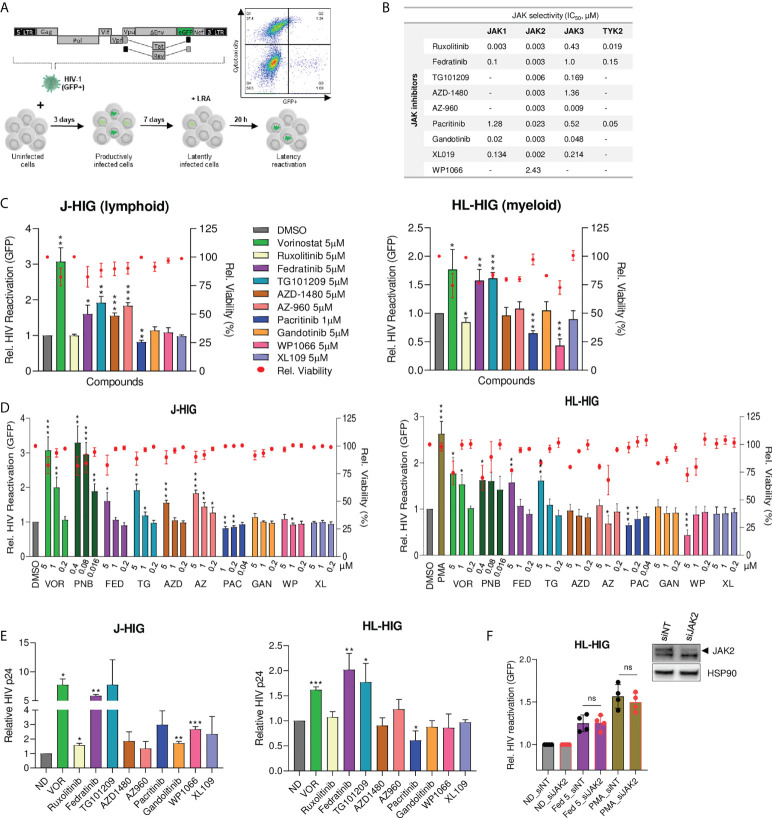
Identification of specific JAK2 inhibitors as latency reversing or latency promoting agents in non-clonal cellular models of HIV-1 latency. **(A)** Generation of non-clonal models of HIV-1 latency for identifying new latency reversal agents (LRA) by flow cytometry. Latently infected models of HIV-1 latency were generated by infecting Jurkat (J-HIG) and HL-60 (HL-HIG) cell lines with the envelope-deficient HIV-1 NL4-3 clone (HIG) encoding GFP. Viral reactivation was measured as the proportion of GFP+ cells 20 hours post incubation with compounds. **(B)** Janus kinase (JAK) selectivity by JAK inhibitors (JAKi) included in the LRA screening. Selectivity refers to 50% inhibitory concentrations (IC_50_) of corresponding enzymatic activities measured in cell free assays. **(C)** HIV-1 latency reactivation capacity of JAKi in J-HIG (left panel) and HL-HIG (right panel) models. Bar plots represent relative HIV-1 reactivation and red dots represent cell viability of treatment conditions normalized to the untreated control (DMSO). **(D)** Expanded screening of JAK2 specific inhibitors in J-HIG (left panel) and HL-HIG (right panel) models. Cells were treated with compounds at the indicated concentrations, as in **(C)**. **(E)** Viral protein production in the J-HIG (left panel) and HL-HIG (right panel) models measured by quantifying HIV-1 CAp24 antigen production 48 h post treatment with indicated compounds using VIP-SPOT assay. **(F)** Effect of JAK2 siRNA knockdown on latency reactivation capacity of LRAs. JAK2 knockdown was confirmed by western blot. Bar plots represent relative HIV-1 reactivation of siRNA JAK2 (siJAK2) and non-targeting (siNT) cells, treated or not with fedratinib at 5 µM or PMA at 100 ng/ml. Data are expressed as mean ± SD of at least three independent experiments. ND, no drug; VOR, vorinostat. ^∗^p<0.05; ^∗∗^p<0.01; ^∗∗∗^p<0.001. not significant (ns) for p>0.05.

To identify new targets for HIV latency reversal or blockade, we developed two non-clonal *in vitro* models of HIV-1 latency based on lymphoid (Jurkat) and myeloid (HL-60) cell lines infected with a GFP-expressing HIV-1 virus (J-HIG and HL-HIG) (**Figure 1A**). Using these models, we assessed the latency reversal capacities of a panel of JAKi with different selectivity indexes for the JAK family (**Figure 1B**). The histone deacetylase inhibitors (HDACi) vorinostat (VOR) and panobinostat (PNB) were used as positive controls for latency reactivation. A specific subclass of selective JAK2i, fedratinib and its structural analogue TG101209, showed potent latency reactivation capacity in both models of HIV-1 latency (2-fold increase, p<0.01), although two other JAKi, AZD-1480 and AZ-960, were able to reverse latent HIV-1 only in the lymphocyte model (**Figure 1C**). Conversely, the JAK2i pacritinib significantly blocked HIV latency reversal in both models (30% decrease, p<0.01), and the pan-JAKi ruxolitinib blocked HIV-1 latency reversal only in myeloid cells, suggesting a putative role of pacritinib as a latency promoting agent (LPA). Expanded dose-response testing of selective JAK2i confirmed screening results, showing significant viral latency reactivation or blockade in a dose-dependent manner (**Figure 1D**). Comparatively similar tendencies in latency reactivation were also observed in the well-established latency cell model ACH2, and in an additional myeloid latency model U-HIG (**Figure S1**). Results in the J-HIG and HL-HIG models were further confirmed by measuring the production of p24 capsid viral protein (CAp24) through viral protein spot (VIP-SPOT) assay, an enzyme-linked ImmunoSpot (ELISpot) approach (23) (**Figure 1E** and **Figure S1**), further suggesting that the use of specific subclass of JAK2i may represent a novel strategy for purging the HIV reservoir.

To determine the contribution of JAK2 as the target cellular factor affecting viral latency, we evaluated the latency reversal capacity of fedratinib in JAK2 depleted cells. Knockdown of JAK2 by siRNA did not affect HIV latency reversal capacity neither in fedratinib nor in phorbol 12-myristate 13-acetate (PMA) treated cells (**Figure 1F**), suggesting that JAK2 function is not affecting HIV-1 latency and thus, pointing to a distinct mechanism of action.

### Fedratinib induces HIV-1 reactivation in the absence of immune activation in CD4+ T lymphocytes *ex vivo*

**Figure 2:**
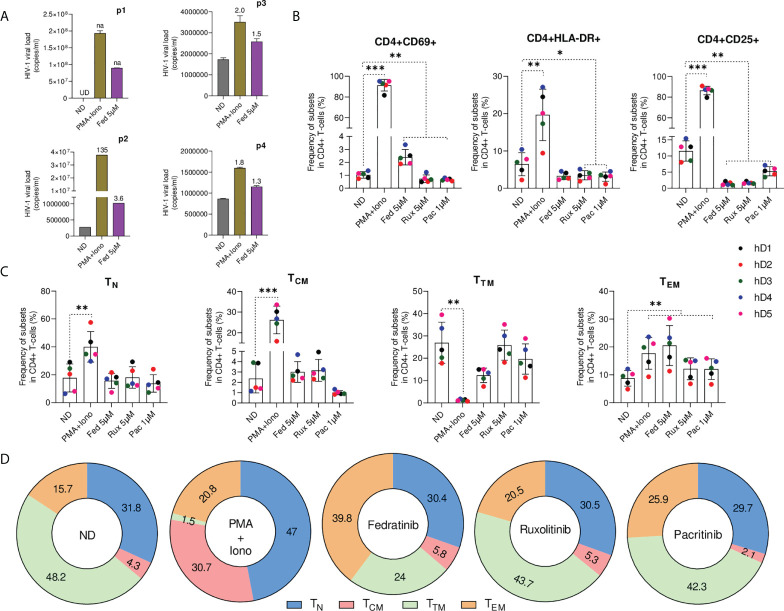
Fedratinib induces HIV-1 reactivation in the absence of immune activation in primary CD4+ T lymphocytes *ex vivo.* **(A)** Quantification of HIV-1 RNA in the cell culture supernatant of isolated CD4+ T cell from ART suppressed HIV-1+ individuals treated with fedratinib. PMA+Iono was used as a positive control for HIV-1 latency reactivation. Absolute HIV-1 copy number (copies/ml) for each HIV-1+ individual are represented as bar plots, and fold changes (FC) relative to the corresponding untreated replicate (ND) are indicated above treatment bars. Undetermined ct values (UD), and FC values if not available (na) are highlighted in the chart. **(B)** Immune cell activation of JAKi-treated CD4+ T cells from healthy donors (n = 5). CD4+ T cells were stained with early (CD69) and late activation (HLA-DR and CD25) markers for flow cytometry 72 h post treatment with compounds. **(C)** Frequency of major CD4+ T cell subsets from JAKi-treated CD4+ T cells from healthy donors. T_N_, naïve T cells; T_CM_, central memory T cells; T_TM_, transitional memory T cells; T_EM_, effector memory T cells. **(D)** Corresponding contribution of individual CD4+ T cell subsets to the major CD4+ T cell pool analysed in **(C)**. Numbers inside sections indicate percentages. Data are pooled from the five healthy donors from **(C)**. Bars show mean ± SD ^∗^p<0.05; ^∗∗^p<0.01; ^∗∗∗^p<0.001.

To evaluate the LRA capacity of JAK2i *ex vivo*, purified resting CD4+ T cells from infected individuals on ART were treated with fedratinib, the best performing JAK2i and viral RNA production in culture supernatant was quantified using an ultra-sensitive nested qPCR (24). Characteristics of study participants are summarized in **Table S1**. In concordance with *in vitro* data, fedratinib was able to *ex vivo* reverse HIV-1 latency to a similar extent than in non-clonal *in vitro* cell line models (**Figure 2A**).

Global T-cell activation is generally detrimental for *in vivo* use, generating interest in LRAs that do not activate T cells (25, 26). To evaluate JAK2i impact on T-cell activation, primary CD4+T cells from uninfected donors were treated *ex vivo* with the selective JAK2i, fedratinib and pacritinib, the pan-JAKi ruxolitinib or PMA+ionomycin as a positive control of immune activation, prior to immunophenotypic characterization by flow cytometry (**Figure S2**). *Ex vivo*, CD4+T cell immune activation was not induced upon JAKi treatment and significant reductions in the frequency of activation markers HLA-DR and CD25 was observed (**Figure 2B**), a condition which has been linked to increased susceptibility of CD4+T cells to productive HIV-1 infection (27). However, in contrast to the other JAKi, treatment with fedratinib slightly induced the early activation marker CD69 but markedly less than the immune activation observed upon treatment with PMA+ionomycin (Fold change FC=2, p=0.002 vs FC=89, p<0.001).

Then, we characterized the relative abundance of the distinct CD4+ T cell subsets treated with JAKi, as distinct CD4+ T cell populations differentially contribute to HIV-1 reservoir (28). Phenotypic characterization of the various CD4+ T-cell populations showed no significant changes in the quiescent naïve (T_N_) and T_CM_ CD4+ T cell phenotype upon treatment with all JAKi relative to the untreated control (**Figures 2C, D**). However, we observed significantly higher enrichment of T_EM_ CD4+ T cell phenotype in fedratinib treated lymphocytes (FC=2.3 vs. untreated control, p<0.01) compared to either pacritinib or ruxolitinib (FC=1.4 vs. untreated control, p<0.01), recapitulating previous observation that T_EM_ phenotype potentiates HIV-1 latency reversal in CD4+ T cells (14). Notably, fedratinib favoured T_EM_ enrichment over T_TM_ CD4+ T cell enrichment, with a concomitant reduction in the T_TM_ phenotype showing a similar trend than PMA+ionomycin, although to a much lesser extent.

### Gene expression changes following JAK2i treatment are comparable to common LRAs

**Figure 3:**
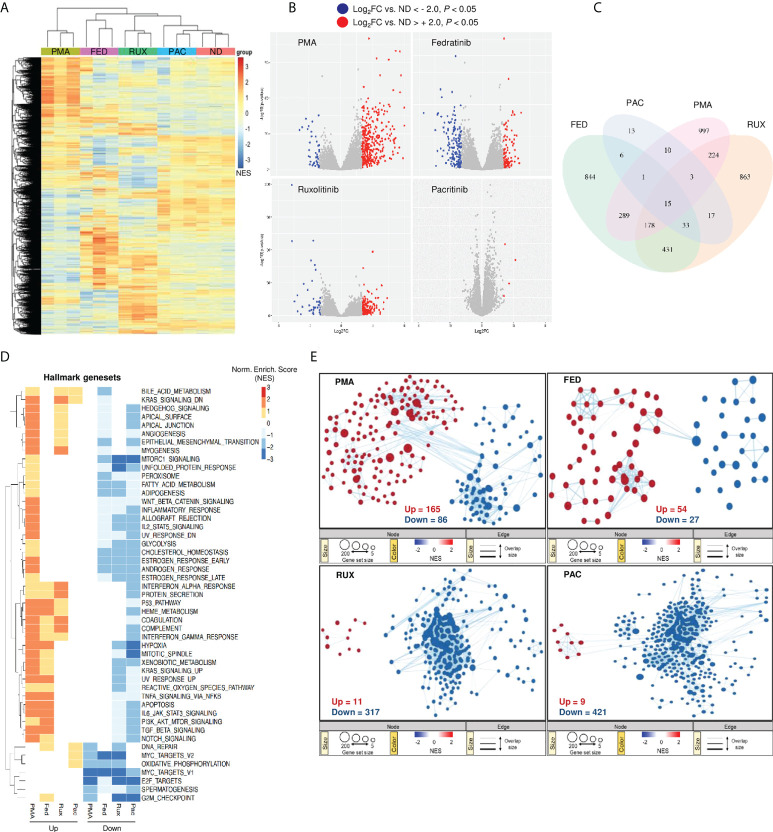
Gene expression changes following fedratinib treatment are comparable to common LRAs. **(A)** Heat map representation of gene expression changes after treatment of HL-HIG cells with JAKi and PMA and untreated control (ND). Drugs were used at concentrations previously shown to be effective at reversing or promoting latency in our model. Heat map for the different treatment conditions was generated by unsupervised hierarchical clustering of significantly expressed genes (p < 0.05). **(B)** Volcano plots of differentially expressed genes (DEG) for each treatment condition relative to the untreated control (ND). DEGs were selected based on Log2 gene expression (Log2FC) and p < 0.05. **(C)** Venn diagram of overlapping DEGs among treatment conditions shown in **(B)**. **(D)** Gene set enrichment analysis (GSEA) for the different treatment conditions using Hallmark genesets. Heatmap visualization of the transcriptional signatures in the distinct treatment conditions was generated using an unsupervised hierarchical clustering of normalized enrichment scores (NES). **(E)** Reactome gene set enrichment map of significantly enriched pathways. The enrichment patterns for the Reactome gene set are highlighted and nodes manually laid out for clarity (full annotation of gene set enrichment map for fedratinib treatment condition is shown in **Figure S4**). Node size represents number of genes, node colour represents significance (NES), and edge thickness represents number of shared genes. Significantly down- or up-regulated genes **(A, B)** and gene sets **(D, E)** are highlighted in blue or red, respectively. Transcriptomic data represents RNA sequencing analysis from three independent experiments.

To delineate the mechanism driving JAK2i-induced effects on HIV-1 latency, whole transcriptome profiling was performed on our myeloid latency *in vitro* model, treated with the LRA fedratinib, the LPA pacritinib, the pan-JAKi ruxolitinib and PMA as a positive control of latency reactivation and immune activation. Hierarchical clustering of all samples using the union of all differentially expressed genes (DEGs) comparing each condition to the control revealed distinct genetic signatures amongst the JAKi (29) (**Figure 3A**). Interestingly, within the JAKi cluster, the LRA fedratinib showed the highest similarity with PMA, the condition with the highest genetic perturbations in contrast to the LPA pacritinib that was identified as the condition with the lowest gene expression changes, being similar to the untreated control (**Figure 3B**). A closer study of overlapping DEGs between the LRAs fedratinib and PMA revealed co-upregulation of several host genes associated with HIV-1 replication cycle, including TNF-α, TNF-associated immediate early response gene 3 (*IER3*) and *CDKN1A* (encoding the cyclin-dependent kinase p21) (**Figure 3C** and **Figure S3**) (30), an effect that was further confirmed in uninfected HL-60 and Jurkat cell lines, linking its expression changes to fedratinib treatment itself (**Figure S3**).

Next, we performed gene-set enrichment analysis (GSEA) using the hallmark gene-sets to elucidate signalling pathways involved in HIV-1 latency modulation by these compounds (31). Enriched gene-sets for each treatment condition relative to the untreated control were annotated and hierarchically clustered with respect to their normalized enrichment scores. Overall, JAKi induced global downregulation of signalling processes, including several pathways involved in cellular metabolism (**Figure 3D**). Unexpectedly, the pathways positively enriched upon fedratinib and PMA treatment (the majority of the genes in these pathways are upregulated), were mainly downregulated in the other JAKi tested. These include gene-sets up-regulated in response to ultraviolet light (UV_RESPONSE_UP), apoptosis (APOPTOSIS) and a group of pathways related to innate immune signalling as TNF-alpha and TGF-beta signalling and IL6-JAK-STAT3 pathway, being contrary to its reported function. Reactome GSEA revealed similar gene-set enrichment patterns between PMA and fedratinib as the hallmark GSEA, further demonstrating the difference between fedratinib and the other JAKi tested (**Figure 3E**). Notably, fedratinib treatment resulted in the least modulation of signalling pathways compared to other JAKi and PMA. In addition, the gene-set enrichment profile of fedratinib mirrored that observed upon PMA treatment with twice as much tendency for up-regulating signalling pathways as PMA (**Figure 3E**, top panel). Moreover, out of all the treatment conditions, only fedratinib significantly enriched the Reactome HIV budding and maturation gene-set (**Figure S4**), confirming its LRA capacity to induce viral protein production (**Figure 1E**).

### Fedratinib upregulates IRF7 expression despite blockade of JAK-STAT signalling and cytokine production

**Figure 4:**
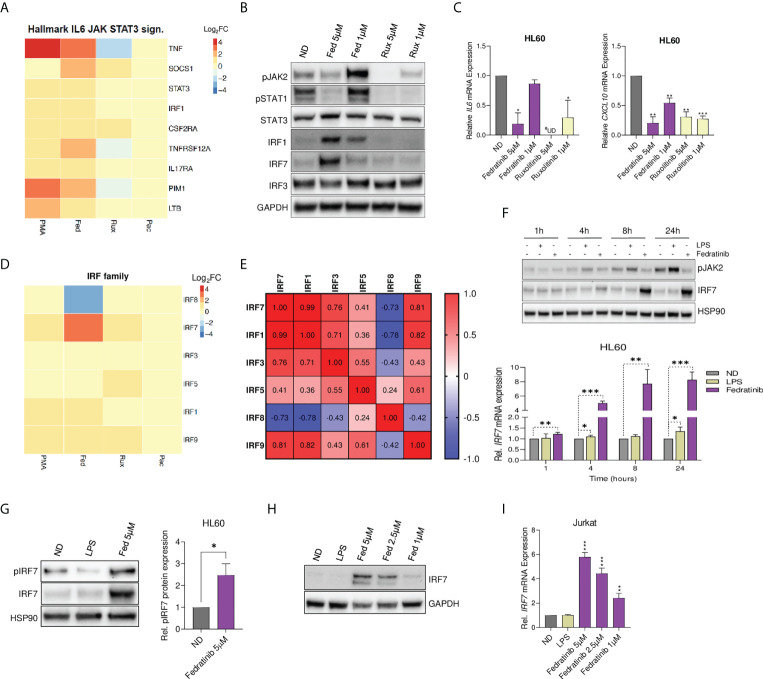
Fedratinib upregulates IRF7 expression despite blockade of JAK-STAT signalling and cytokine production. **(A)** Gene expression of leading-edge genes from the Hallmark IL6-JAK-STAT3 signalling gene set. Fedratinib treatment condition was used as a reference set for leading-edge genes selection and ranking. **(B)** Evaluation of JAK-STAT signalling pathway at protein level in JAKi-treated HL60 cells using western blot analysis. GAPDH was used as a loading control. **(C)** Gene expression of *IL6* and *CXCL10* following treatment with JAKi. Relative mRNA expression was measured by quantitative RT-PCR and normalized to GAPDH. **(D)** Heatmap showing all differentially expressed interferon regulatory factors (IRF) from whole transcriptome analysis. **(E)** Spearman correlation matrix for differentially expressed IRF. Negatively and positively correlated gene expression of IRF in the transcriptomic data are shown in blue and red, respectively. **(F)** Western blot analysis showing time dependence of IRF7 induction by fedratinib despite consistent blockade of JAK2 phosphorylation. LPS treatment was used as a positive control for the activation of the JAK-STAT pathway (induction of pJAK2), and HSP90 as a loading control. Induction of IRF7 gene expression was confirmed by qRT-PCR. **(G)** IRF7 activation measured by protein expression of phosphorylated IRF7 (pIRF7) in HL60 cells treated with either fedratinib or LPS for 24 h A representative experiment is shown. Bar plots represent the mean quantification of pIRF7 bands obtained by densitometry analysis of three independent experiments. Values were normalized to that of Hsp90 used as a loading control and relativized to the untreated condition (ND). **(H, I)** Dose-response induction of IRF7 expression upon 24 h fedratinib treatment in Jurkat cells by Western blot analysis **(H)** and qRT-PCR **(I)**. Data are expressed as mean ± SEM of at least three independent experiments. ^∗^p<0.05; ^∗∗^p<0.01; ^∗∗∗^p<0.001. ^#^UD - undetermined cycle threshold (below limit of detection).

Taking into account the differences observed between fedratinib and other JAKi, and the distinct observed effects on JAK-STAT signalling pathway, we conducted a more detailed evaluation of the pathway. Examination of leading-edge genes of the JAK-STAT hallmark gene-set indicated that none of the JAKs was enriched, but instead a significant upregulation of STAT3 and IRF1 was observed (**Figure 4A**). To confirm the RNA-Seq data, the modulation of JAK-STAT signalling pathway was validated in cells treated with either fedratinib or ruxolitinib. As expected, all JAKi induced a potent downregulation of pJAK2, pSTAT1 (**Figure 4B**) and IFN-stimulated genes as IL6, CXCL10 and IL8 in multiple cell lines (**Figure 4C**, **Figure S5A**), indicating efficient blockade of the JAK-STAT signalling pathway. Similar results were obtained in A549 dual cells, where fedratinib blocked IFN-sensitive response element (ISRE) and NF-κB induced transcription, measured by corresponding reporter genes, despite enrichment of the TNFα signalling pathway (**Figure S5B-D**). However, fedratinib upregulated the expression of the interferon regulatory factors IRF1 and IRF7 (**Figure 4B**), although only IRF7 expression was differentially modulated, depending on the JAKi used, ranging between no change to 8 FC from pacritinib to fedratinib, respectively (**Figure 4D**). We also observed that the expression of IRF7 positively correlated with other transcriptional activating IRFs, i. e., IRF1, IRF3, IRF5 and IRF9, but not with the transcriptional repressor IRF8 (**Figure 4E**) (32).

According to the classical model of JAK-STAT signalling pathway, transcriptional activity of the IRFs is induced downstream of crucial immune sensors for transcription of type I IFN, that subsequently lead to cytokine and IFN-induced phosphorylation of the JAKs and STATs, leading to the transcription of ISGs and other target genes. Hence, we further evaluated the dependency of fedratinib-induced IRF7 expression and classical JAK-STAT signalling over time, using lipopolysaccharide (LPS) as a positive control for activation of the JAK-STAT signalling pathway. Interestingly, LPS failed to induce IRF7 expression despite clearly inducing activation of the JAK-STAT pathway (phospho-JAK2 induction). On the contrary, fedratinib treatment significantly upregulated IRF7 expression despite blocking JAK2 activation in a time-dependent manner (**Figure 4F**). Interestingly, fedratinib also upregulated IRF7 phosphorylation, indicative of increased IRF7 activation which allows translocation to the nucleus and at its turn that might impact viral transcription (**Figure 4G**). Significant induction of IRF7 expression was also observed in a dose-response treatment of fedratinib in the lymphoid Jurkat cell line (**Figures 4H, I**). Overall, our results suggest that fedratinib induces HIV-1 reactivation through an IFN-independent regulation of IRF7 transcription and activation.

### Induction of IRF7 expression positively correlates with latency reversal modulation

**Figure 5:**
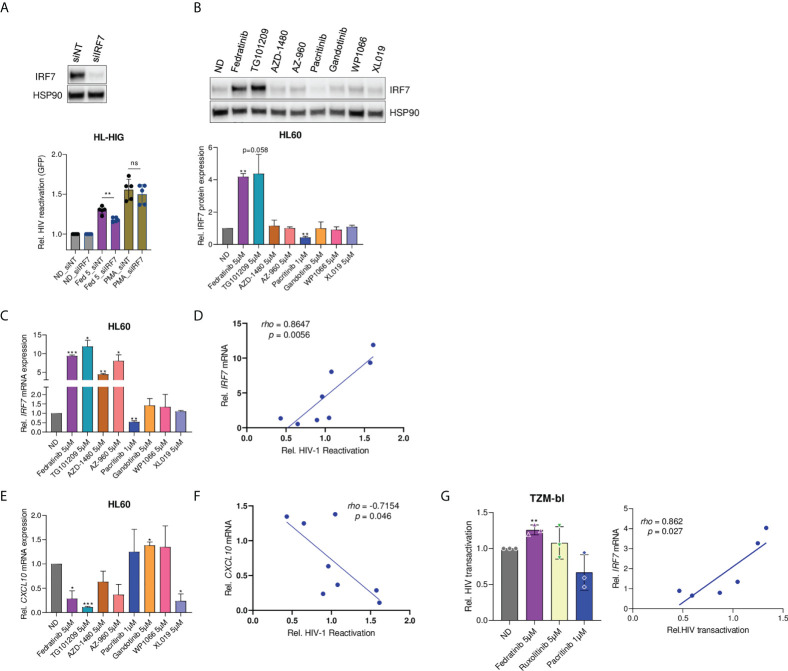
Modulation of IRF7 expression correlates with latency reversal capacity. **(A)** Effect of IRF7 siRNA knockdown on latency reactivation capacity of LRAs. IRF7 knockdown was confirmed by western blot (pictograph). Bar plots represent relative HIV-1 reactivation of IRF7 knockdown (siIRF7) and non-targeting (siNT) control cells, treated or not with fedratinib at 5 µM or PMA at 0.1µg/ml. **(B)** IRF7 protein expression in HL60 cells treated with the distinct JAK2i. A representative experiment is shown. Bar plots represent the mean quantification of bands obtained by densitometry analysis of 3 independent experiments. Values were normalized to that of Hsp90 used as a loading control and relativized to the untreated condition (ND). **(C-E)** Gene expression of *IRF7* and *CXCL10* in JAK2i -treated HL-60 cells. Relative mRNA expressions are expressed as fold-change differences in expression of gene-of-interest relative to GAPDH gene. **(D, F)** Correlation plots of *IRF7* and *CXCL10* gene expression **(C, D)** versus HIV-1 latency reversal capacity of JAK2i-treated cells. **(G)** Fedratinib induces Tat-dependent viral expression of HIV-1 LTR promoter and correlated with IRF7 expression. HeLa TZM-bl cells were transfected with Tat-expressing plasmid for 24h. HIV-1 transactivation was measured by a luciferase-based assay 20 hours post treatment with compounds. Left panel, relative Tat-induced HIV-1 transactivation upon JAK2i treatment. Right panel, correlation between HIV-1 transactivation and IRF7 expression (see also **Figure S5F**). Values were normalized to the untreated control. Data are expressed as mean ± SD of at least three independent experiments. ^∗^p<0.05; ^∗∗^p<0.01; ^∗∗∗^p<0.001. not significant (ns) for p>0.05.

To further investigate IRF7 role in latency reversal, IRF7 expression was knocked-down by siRNA in myeloid HIV-1 latently infected cells. IRF7 knockdown significantly impaired the HIV-1 latency reversal capacity of fedratinib (p = 0.005, **Figure 5A**), confirming the key role of IRF7 expression. Then, modulation of IRF7 expression was evaluated in the extended panel of JAK2i, showing significant upregulation only in cells treated with the other JAK2i acting as LRAs, in contrast to the significant downregulation of IRF7 by the LPA Pacritinib, at both protein (**Figure 5B**) and mRNA expression level (**Figure 5C**). Indeed, the latency-reversing capacity of the distinct JAK2i tested, directly correlated with IRF7 expression (rho=0.86, p=0.0056, **Figure 5D**). In contrast, treatment with JAK2i-LRAs significantly blocked CXCL10 production (**Figure 5E**), providing additional proof of the independent role of anti-inflammatory signalling and IRF7 induction and indicating an inverse correlation with latency reactivation in our model (rho=-0.715, p=0.046, **Figure 5F**). Overall, these results suggest a direct effect of IRF7 expression on HIV-1 latency reversal, putatively through LTR transcriptional induction.

Regarding the viral proteins studied till date, HIV-1 Tat has attracted more attention in viral latency because it potently plays a crucial role in viral transcription regulation. Thus, to further investigate the role of IRF7 in HIV-1 viral transcription, an HIV-1 Tat transactivation assay was performed in HeLa TZM-bl cells. TZM-bl cells harbour an integrated copy of HIV-1 LTR, controlling luciferase reporter gene expression, which mimics a latent integrated provirus. First, TZM-bl cells were validated as an appropriate model for measuring Tat-dependent viral reactivation by dose-dependent transfection of a Tat expression plasmid in the presence of PMA, as a positive control of transcription induction (**Figure S5E**). Then, Tat expressing TZM-bl cells were treated with JAK2i to determine the role of fedratinib-induced IRF7 expression on Tat-dependent viral reactivation. IRF7-dependent latency reactivation was observed upon treatment with JAK2i, with significant induction in Tat-mediated HIV-1 transcription by fedratinib in contrast to pacritinib (p=0.003 vs p=0.079, **Figure 5G** and S5F). More importantly, IRF7 expression levels significantly correlated with Tat-mediated transactivation (**Figure 5G**, right panel), further demonstrating the role of IRF7 in Tat-mediated HIV-1 transcription.

### IRF7 is a general regulator of HIV-1 latency

**Figure 6:**
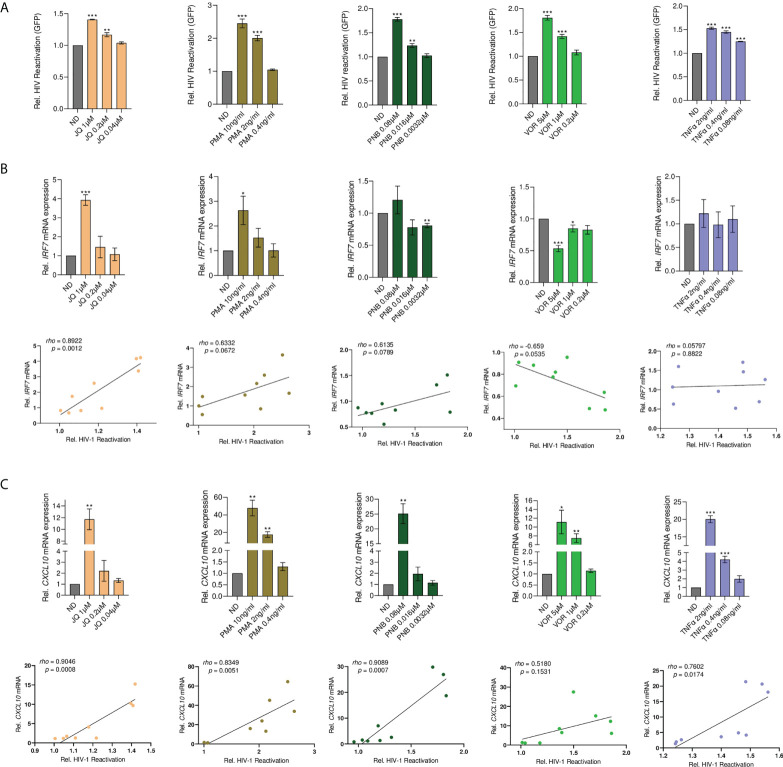
Latency reactivation by common LRAs positively correlates with the induction of ISG. **(A)** Dose-response latency reversal activity by distinct well-described LRAs in a model of HIV-1 latency (HL-HIG). Bar plots represent relative HIV-1 reactivation normalized to the untreated control (ND). **(B)** Top panels, *IRF7* gene expression upon treatment with the indicated LRAs. Relative mRNA expressions are expressed as fold-change differences in expression of gene-of-interest relative to GAPDH gene. Bottom panels, Pearson correlation plots of HIV latency reversal activity versus *IRF7* gene expression. **(C)** Top panels, *CXCL10* gene expression upon treatment with the indicated LRAs. Relative mRNA expressions are expressed as fold-change differences in expression of gene-of-interest relative to GAPDH gene. Bottom panels, pearson correlation plots of HIV latency reversal activity versus *CXCL10* gene expression. All statistical comparisons were performed with Student’s t tests. *p<0.05; **p<0.01; ***p<0.001. Data are expressed as mean ± SD of at least three independent experiments.

To investigate whether IRF7 plays a more general role in HIV-1 latency, we screened a panel of common LRAs for IRF7 dependency, including the HDACi vorinostat (VOR) and Panobinostat (PNB), the BET bromodomain inhibitor JQ1, TNF-α, and PMA. As expected, all tested LRAs showed significant latency reactivation capacity in the HL-HIG model (p<0.001, **Figure 6A**). JQ1 and PMA treatment significantly upregulated IRF7 expression in a dose-dependent manner and in the case of JQ1, positively correlated with its latency reactivation capacity (rho=0.892, p=0.001 for JQ1, **Figure 6B**). Conversely, no effect was observed for TNF-α or PNB, and the HDACi VOR significantly blocked IRF7 induction in the HL60 model, being negatively correlated with its latency reactivation capacity. Importantly, all common LRAs significantly induced CXCL10 production with a strong positive correlation, in contrast to the JAK2i-LRA (**Figure 6C**). Altogether, these data suggest that although IRF7 role in latency reversal is not common to all LRAs, it is not limited to JAK2i, indicating a novel but yet unrecognized mechanism that might be relevant for the development of novel therapeutic strategies directed towards HIV cure.

### Fedratinib is a potent antiviral agent in acute HIV infection

**Figure 7:**
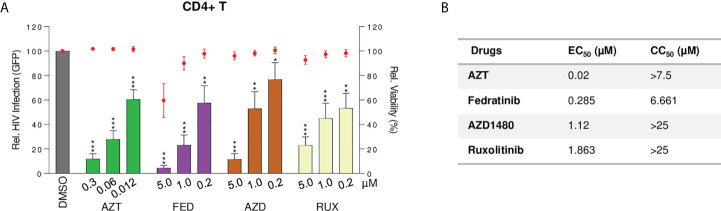
Fedratinib is a potent antiviral agent in acute HIV infection, specifically targeting multispliced HIV RNA transcripts. **(A)** Antiviral activity of JAKi in acute HIV infection of activated CD4+T cells from healthy donors (n = 3). HIV-1 infection was measured as proportion of GFP+ CD4+ T cells after infection with GFP-encoding NL4-3 env-deficient HIV-1 clone, 48 h post treatment with compounds at the indicated concentrations. Bar plots represent relative HIV infection and dot plots cell viability of corresponding treatment conditions normalized to the untreated control (DMSO). **(B)** EC_50_ and CC_50_ values of JAKi in acute HIV-1 infection model. Values were calculated from **(A)**. EC_50_, effective concentration required to block HIV-1 replication by 50% and CC_50_, concentration required to induce 50% of cell death in cell culture. (Values are expressed as mean ± SD. **p<0.01; ***p<0.001.

Several JAKi, including ruxolitinib and tofacitinib, are potent inhibitors of HIV-1 replication (17, 18, 33). This observation raises the possibility that fedratinib, besides reversing HIV-1 latency, might also block new HIV-1 infections, a highly desired effect in a clinical setting. Thus, we evaluated the antiviral activity of selective JAK2i fedratinib and AZD1480, and the pan-JAKi ruxolitinib, in acute HIV-1 infection in primary CD4+T cells from healthy donors (n=3). Fedratinib and other JAKi potently blocked HIV-1 replication in acute HIV-1 infection (**Figure 7A**). Interestingly, fedratinib was the most potent antiviral agent of all the JAKi tested (EC_50 =_ 0.285µM, **Figure 7B**).

## Discussion

Current ART can achieve long-term viral suppression, although with several limitations, including the inability to cure HIV infection due to the presence of latent reservoirs and failure to address HIV-driven inflammation. Over the last years, various LRAs have been explored as putative cure strategies, with several PKC activators and HDAC inhibitors undergoing clinical trials for the elimination or reduction of latent reservoirs, but with mostly unsatisfactory curative effects (3, 34, 35). Hence, the identification of new LRAs capable of reversing HIV-1 latency without causing global T-cell activation and reducing inflammatory signalling as the specific subclass of JAK2 inhibitors here characterized, are of key importance to achieve a functional cure. Moreover, functional characterization of latency reversal activity of JAK2i allowed the identification of IRF7 as a new common cellular factor controlling HIV-1 latency in both lymphoid and myeloid cell lines.

Current LRAs are mainly designed to reactivate the HIV-1 provirus in CD4+ T cells, but their ability to abolish viral latency in myeloid-derived cells is largely unknown. Moreover, the resistance of macrophages to HIV-1 mediated killing and the presence of infected macrophages in immune-privileged regions, including the central nervous system, may pose a barrier to eliminating infected cells by current “shock and kill” strategies (28). Recent advances have highlighted the importance of myeloid reservoirs as sanctuaries of HIV persistence (28); however, current techniques are primarily based in T cells and have not been able to comprehensively and specifically map the myeloid HIV reservoir, due to its much smaller size and the more widely dispersion throughout the body, highlighting the need of novel *in vitro* and *ex vivo* experimental models. Thus, our initial *in vitro* screening was performed in parallel in non-clonal myeloid and lymphoid models of HIV-1 latency, which allowed the identification of a specific subclass of JAK2 inhibitors with broad and potent latency reactivation activity. JAKi are FDA-approved drugs for the treatment of myelofibrosis and rheumatoid arthritis. The distinct JAKi differ in their selectivity for the JAKs, JAK1-3 and TYK2, with demonstrable phenotypic differences in their different modes of action (29). Interestingly, reactivation potential was not common to all JAK inhibitors tested, putatively due to their distinct selectivities and more relevant, to the effects exerted over other host signalling pathways (29, 36). Previous studies have identified JAK1/2i as antiviral agents against HIV infection, blocking both latent and productive infection, in line with our findings for the pan-JAK inhibitor ruxolitinib and JAK2i pacritinib (17, 33). In contrast, the JAK2i fedratinib and its analogue TG101209 significantly induced HIV-1 reactivation both *in vitro* and *ex vivo*, an effect that was independent of their inhibitory activity on the JAK-STAT pathway and subsequent anti-inflammatory properties. Indeed, we found that *in vitro* treatment of primary lymphocytes with fedratinib did not induce major changes in immune cell activation or CD4+ T cell populations, except for enrichment in the proportion of CD4+ T effector memory cells. Emerging evidence suggests that agents impairing differentiation into T effector memory phenotype *in vivo* block latency reactivation (37), highlighting the importance of effector memory cells as an important latent reservoir for eradication strategies within the context of “shock and kill”.

The identification of FDA-approved compounds such as fedratinib that can purge HIV-1 reservoir in distinct cell types but also harbour potent immunomodulatory properties, specifically counteracting hyperinflammatory responses and restoring normal immune signalling, represents a valuable asset for HIV cure strategies (2). These desirable attributes prompted us to characterize its mode of action to identify the cellular pathways underlying its latency reactivation potential. Indeed, whole transcriptome profiling and pathway analysis confirmed the singularity of fedratinib compared to other JAKi. Interestingly, fedratinib and PMA, but not the JAKi non-LRAs ruxolitinib and pacritinib, similarly upregulated pathways associated with apoptosis and several immune-related signatures. More importantly, fedratinib was the inhibitor presenting the lowest number of altered gene sets in the pathway analysis, suggesting the existence of fewer undesired off-target interactions. Small molecule drugs interact with unintended, often unknown, biological targets, and these off-target effects may lead to both preclinical and clinical toxic events, thus, the fewer off-targets, the better for its clinical applications.

The mechanisms for the establishment, maintenance and reactivation of HIV-1 latency mainly operate at the transcriptional level by both viral (38–41) and host machinery, usually through chromatin modification and epigenetic regulations (40, 42–44). Thus, based on pathway analyses, in depth evaluation of specific candidate genes responsible for fedratinib latency reactivation activity focused on pathways controlling host cell transcription, leading to the identification of IRFs as putative targets, and among them IRF7, showing the most significant change in gene expression, both in lymphoid and myeloid cells. IRFs are a family of transcription factors that play pivotal roles in many aspects of the immune response, including immune cell development and differentiation and regulating responses to pathogens. Among them, IRF3, IRF5, and IRF7 are critical to the production of type I interferons downstream of pathogen recognition receptors that detect viral RNA and DNA. IRF7 is a multifunctional transcription factor (45) that exhibits broader DNA binding specificity than other IRFs (46), and its expression and activation is also more tightly regulated (45).

At first glance, the upregulation of IRF7 expression and activation despite the effective blockade of the IFN-induced JAK-STAT signalling pathway may seem counterintuitive. However, our data clearly pointed towards a new, as yet unrecognized role of IRF7, not directly linked to its canonical and well-described role as a master regulator of IFN expression (47). In agreement with our data, an IFN-independent regulation of IRF7 transcription has already been described in the literature (47), representing an alternative mechanism for IRF7 activation, albeit significantly less studied than the IFN-mediated classical signalling pathway. We provide several compelling evidences demonstrating IRF7 role in lymphoid and myeloid-derived experimental models of HIV latency, including (i) specific impairment of fedratinib latency reactivation activity upon IRF7 knockdown, (ii) direct correlation of IRF7 expression levels and HIV latency reactivation and (iii) correlation between Tat-mediated HIV-1 transcription induced by JAK2i and IRF7 upregulation. Indeed, upregulation of IRF7 expression has been previously identified in cells treated with BRD4 inhibitors acting as LRA in J-Lat latently infected HIV-1 model (48). In line with this data, we also observed that IRF7 expression directly correlated with HIV-1 reactivation in response to distinct LRAs, including PMA, the BRD4 inhibitor JQ1 and the HDACi Panobinostat, in addition to specific JAK2i. Moreover, upregulation of IRF7 expression was independent of IFN-related gene expression as all LRAs except those blocking JAK function significantly up-regulated ISG expression, providing further proof of the independent role of IRF7 in HIV-1 latency reactivation. Furthermore, through genome-wide quantitative trait locus analysis in chronic HIV-1 infected individuals, IRF7 has been identified as a host genetic factor affecting the size and transcriptional activity of HIV-1 reservoirs, further supporting the role of IRF7 in HIV-1 latency (49).

Overall, we have identified and characterized the JAK2i, fedratinib, as a novel LRA. Several attributes distinguish fedratinib as an excellent candidate for HIV cure strategy, including the demonstrated capacity in reversing HIV-1 latency without causing global T-cell activation, reducing inflammatory signalling, and high specificity in the modulation of a limited number of signalling pathways. Moreover, functional characterization of fedratinib latency reactivation capacity revealed new molecular determinants of HIV-1 latency maintenance and reactivation, specifically IRF7. Therefore, mechanistic insights into the molecular basis of the divergent roles played by IRF7 in host protection against viruses warrant further exploration in future studies.

## Materials and methods

### Primary cultures and cell lines

Buffy coats from healthy donors were purchased from the Catalan Banc de Sang i Teixits (http://www.bancsang.net/en/index.html). The buffy coats received were totally anonymous and untraceable and the only information given was whether or not they have been tested for diseases. All donors provided informed consent at the time of blood collection. All methods were carried out in accordance with relevant guidelines and regulations and to the ethical principles suggested in the Declaration of Helsinki.

Peripheral blood mononuclear cells (PBMC) from buffy coats of healthy donors and PWH study participants were obtained by Ficoll-Paque density gradient centrifugation and used for fresh purification of CD4+ T lymphocytes (StemCell Technologies). Purity of the CD4+ T lymphocyte population was confirmed by flow cytometry. CD4+ T lymphocytes were kept in complete RPMI culture medium (RPMI 1640 medium supplemented with 10% heat-inactivated fetal bovine serum (FBS; Gibco), 100 U/ml penicillin, 100 μg/ml streptomycin (Gibco, Life Technologies)), hIL-2 (6.5 IU/ml, Roche) and stimulated (donor) or not (PWH study participants) for 3 days with PHA (4 µg/ml; Sigma-Aldrich).

The human cell lines HL60, Jurkat, U937, MT-4, HEK293T, ACH2 and TZM-bl cells were obtained from the AIDS Reagent Program, National Institutes of Health (Germantown, MD). HL60, Jurkat, U937, MT-4 and ACH2 cells were cultured in complete RPMI culture medium (RPMI 1640 medium supplemented with 10% FBS, 100 U/ml penicillin and 100 μg/ml streptomycin). HEK293T and TZM-bl cells were cultured in Dulbecco’s modified Eagle’s medium (DMEM), supplemented with 10% FBS, 100 U/ml penicillin and 100 μg/ml streptomycin.

A549-Dual™ hACE2-TMPRSS2 cells (*In vivo*Gen) were cultured in complete DMEM as described above in addition to the following antibiotics: 10 µg/ml of Blasticidin, 100 µg/ml of Hygromycin, 0.5 µg/ml of Puromycin, and 100 µg/ml of Zeocin, (*In vivo*gen). A549-Dual™ cells are adherent epithelial cells derived from the human A549 lung carcinoma cell line with a stable integration of two inducible reporter constructs for studying the expression of NFκB and interferon regulatory factor (IRF) pathways.

All cell cultures were maintained at 37°C in a 5% CO_2_ incubator.

### Patients and samples

This study sampled a cohort of chronically infected HIV-1 positive individuals attending Hospital Germans Trias i Pujol, Badalona, Spain. Study participants were included if the individuals were older than 18 years old and had been on suppressive ART with undetectable plasma HIV-1 RNA levels (<40 copies/ml) for a minimum of two years. All participants were males (n=4). The median age of the participants was 45y (range 38-56y). Frozen PBMCs (isolated as described below and cryopreserved) from PWH visiting our clinic were used. Immunological and virological characteristics from all participants are found in **Table S1**. All participants in the study provided informed consent, and the work was approved by the Scientific Committee of Fundació IrsiCaixa and the Ethics Committee of Hospital Germans Trias i Pujol (Ref. CEI PI-18–021).

### Viral strains

The envelope-deficient HIV-1 NL4-3 clone (HIG) encoding internal ribosome entry site (IRES)-green fluorescent protein (GFP) (NL4-3-GFP) (50) was pseudotyped with vesicular stomatitis virus G protein (VSV-G) by cotransfection of HEK293T cells using polyethylenimine (Polysciences) as previously described (51, 52). HIV-1 stock of the fully replicative NL4-3 clone (NL4-3) was grown in lymphoid MT-4 cells. Three days after transfection, supernatants were harvested, filtered and stored at -80°C. Viral stocks were concentrated using Lenti-X concentrator (Clontech). Viruses were titrated by infection of TZM-bl cells followed by GFP quantification by flow cytometry or qPCR for HIG or NL4-3, respectively.

### Generation of non-clonal HIV latently infected cells and viral reactivation assays

Latently infected cells (J-HIG, HL-HIG and U-HIG) were generated by infecting lymphoid (Jurkat) and myeloid (HL60 and U937) cells with HIG virus and maintained in culture for 10 days to allow for the attrition of productively infected cells as previously described (53). HIV-1 reactivation was measured as the percentage of GFP + cells by flow cytometry 20 h post incubation with the compounds relative to untreated control (DMSO). The capacity of known and putative LRAs to induce HIV-1 expression was confirmed in latently infected ACH2 cells by measuring intracellular HIV CAp24 by flow cytometry 48 h after treatment.

For evaluation of cell death, cells were stained for 30 min with LIVE/DEAD™ Fixable Near-IR Dead Cell Stain Kit (Invitrogen, Thermo Fischer Scientific) in PBS according to manufacturer’s instructions. Alternatively, viable cells were identified according to forward and side laser light scatter flow cytometry analysis. Cells were washed and fixed in 1% formaldehyde before the analysis. Flow cytometry assays were performed in a FACS LSR II or a FACSCanto II flow cytometer (BD Biosciences). The data was analyzed using the FlowJo software (BD Biosciences).

### Compounds and drug treatments

PMA (Sigma-Aldrich) was used at 0.4-100 ng/ml and ionomycin (Sigma-Aldrich) was used at a concentration of 100 ng/ml. 3-azido-3-deoxythymidine (zidovudine; AZT) (Sigma-Aldrich) was used at 10 µM, raltegravir at 5 µM and efavirenz at 0.32 µM were obtained from the NIH AIDS Research and Reference Reagent Program. TNFα (Merck) was used at a concentration of 0.08-2 ng/ml and LPS at 100 ng/ml (Merck). Fedratinib, TG101209, AZD1480, AZ960, gandolitinib, WP1066, XL109, ruxolitinib, momelotinib, JQ1 were used at a concentration of 0.2-5 µM, pacritinib at 40 nM-1 µM and Q-VD-Oph at 10 µM (all from Selleckchem). Vorinostat (SAHA) was used at a concentration of 0.2-5 µM (Prochifar srl, Italy) and panobinostat at 3.2-400 nM (LC Laboratories).

### VIP-SPOT assay

ELISpot plates (Immobilon-P polyvinylidene difluoride membrane, Cat#MSIPS4W10; Millipore) were coated in advance with 10mg/ml capture antibody (mouse monoclonal antibody to HIV p24; clone 39/5.4A, Cat#ab9071; Abcam). On the day of cell culture, the wells were washed with sterile PBS and incubated with 100ml of a blocking solution containing 5% bovine serum albumin (BSA) (MACS BSA stock solution, Cat#130-091-376; Miltenyi) in PBS, for at least 30 min at room temperature. This was followed by additional washing of wells and replacement with culture medium containing HL-HIG or J-HIG cells at 9 x 10^3^ cells per well and compounds. After 48 h of incubation at 37°C in a 5% CO2-humified atmosphere, the plates were developed as described elsewhere (23). After drying, the spots were counted using an automated ELISpot reader unit (Cellular Technology Limited, Shaker Heights, OH).

### *Ex vivo* reactivation of primary CD4+ T cells from PWH

Purified CD4+ T lymphocytes from HIV-1+ participants were kept in complete RPMI culture medium and preincubated with the pan-caspase inhibitor Q-VD-Oph (10 µM, Sigma-Aldrich) for 2 h. To evaluate the latency reactivation capacity, half a million CD4+ T lymphocytes were cultured with PMA + ionomycin or fedratinib at the indicated concentrations (5 µM fedratinib, and 50 ng/ml PMA + 1 µg/ml ionomycin). Cells were cultured in the presence of antiretrovirals (efavirenz, zidovudine and raltegravir) and maintained in 10 µM Q-VD-Oph for 72 h.

Freshly collected cell-culture supernatant were centrifuged for 1 hour at 25 000 g to pellet HIV particles. HIV-1 reactivation was determined by quantification of viral RNA in the supernatant as previously described (24). Briefly, viral RNAs were extracted using Viral RNA/DNA Mini kit (Invitrogen) and quantified using a nested real-time reverse transcription-polymerase chain reaction (RT-PCR). A Superscript III One-Step RT-PCR system (Invitrogen) was used to generate and pre-amplify viral RNA with the following primers: ULF1 (forward) 5’- ATG CCA CGT AAG CGA AAC TCT GGG TCT CTC TDG TTA GAC - 3’; UR1 (reverse) 5’- CCA TCT CTC TCC TTC TAG C -3’. The following cycling conditions were used: reverse transcription at 50°C for 30 min, denaturation at 94°C for 2 min, 16 cycles of amplification (94°C 15 s, 55°C 30 s, 68°C 1 min) and final elongation at 68°C for 5 min. Preamplified products were subjected to nested real-time PCR with the following primers and probe: LambdaT (forward) 5’- ATG CCA CGT AAG CGA AAC T - 3’; UR2 (reverse) 5’ - CTG AGG GAT CTC TAG TTA CC - 3’; UHIV Taqman 5’- 56-FAM/CAC TCA AGG/ZEN/CAA GCT TTA TTG AGG C/3IbkFQ/- 3’ on a QuantStudio 5 PCR system (Applied Biosystems). Serial dilutions of the HIV-1 NL43 strain were run in parallel with each experiment for the quantification of viral RNA.

### Immunophenotypic characterization of PBMCs by flow cytometry

Purified CD4+ T lymphocytes from freshly processed PBMCs isolated from healthy donors were stained with CD4+T cell phenotype defining markers for flow cytometry 72 h post treatment with compounds: CD4-BV786, CD45RA-PE, CCR7-BV510 (Biolegend); CD3-FITC, CD27-400 (BD Biosciences). Immune activation was determined using CD69-BV650, CD25-APC (Biolegend) and HLA-DR-PeCy7 (BD Biosciences). Immunophenotyping of CD4+ T cell population identification was performed based on the following gating strategy (**Figure S2**): CD4 + T cell populations were defined based on the following expression combinations gated on the live singlet CD3+CD4+ lymphocytes: T naïve T_N_ (CD45RA+CCR7+CD27+), T central memory T_CM_ (CD45RA-CCR7+CD27+), T transitional memory T_TM_ (CD45RA-CCR7-CD27+) and T effector memory T_EM_ (CD45RA-CCR7+CD27-) (13). Immune activation markers HLA-DR+, CD25+ and CD69+ CD4+ lymphocytes were also gated on the live singlet CD3+CD4+ lymphocytes. Cells were washed and fixed in 1% formaldehyde before the analysis. Flow cytometry assays were performed in a FACS LSR II or a FACSCanto II flow cytometer (BD Biosciences). The data was analyzed using the FlowJo software (BD Biosciences).

### RNA interference

Cells were transfected using Amaxa Cell line Nucleofector Kit V (VCA-1003, Lot F-13862, Lonza) in a 6-well plate following manufacturer instructions for RNAi transfections. Briefly, 20 pmol of the corresponding siRNA was nucleofected in 3 x 10^6^ of the corresponding cell line. RNA and protein lysates were collected 24 h post-transfection. siRNAs used for transfection were ON-TARGETplus Non-targeting siRNA Pool (D-001810-10-05), human JAK2 siRNA-SMARTpool (L-003146-00- 0005), and human IRF7 siRNA-SMARTpool (L-011810-00-0005) all from Dharmacon, Waltham, USA.

### Quantitative RT-polymerase chain reaction (qRT-PCR)

Total RNA was extracted using NucleoSpin RNA II kit (740955, Macherey-Nagel) or the Maxwell^®^ HT simplyRNA Kit (REF: AX2420, LOT: 0000410025, Promega) and Total DNA was extracted using Magmax DNA multi-sample ultra 2.0 kit (A45721, appliedbiosystems) on a KingFisher™ Flex Purification System (Thermofisher Scientific) as recommended by the manufacturer. Reverse transcription was performed using the PrimeScript™ RT-PCR Kit (RR036A, Takara) following manufacturer instructions. mRNA levels of all genes were measured by two-step quantitative RT-PCR and normalized to GAPDH mRNA expression using the DDCt method. Primers and DNA probes were TaqMan Gene expression assays from Thermofisher Scientific (Cat#433182: Hs00266705_g1-GAPDH, Hs01014809_g1-*IRF7*, Hs00171042_m1-*CXCL10*, Hs00174131_m1-*IL6*, Hs00174103_m1-*IL8*, Hs00355782_m1-*CDKN1A*, Hs00174128_m1-*TNFα*, and Cat#4351372: Hs00931699_m1-*CIITA*).

### RNA-sequencing and library preparation

Cellular RNA was extracted from the latent model of HIV-1 infection, HL-HIG for RNA sequencing using Maxwell^®^ HT simplyRNA Kit (Promega) on a KingFisher™ Flex Purification System (Thermofisher Scientific). RNA-sequencing samples were prepared in biological triplicates. Determination of RNA integrity (RIN) was performed using Agilent RNA 6000 Nano Kit (Cat#5067-1511) and Agilent 2100 Bioanalyzer System. After quality control check, RNA library was constructed using Illumina TruSeq Stranded mRNA LT Sample Prep Kit and sequencing was performed using NovaSeq 6000 System with 150 bp paired-ends reads.

### Transcriptomic analysis

Transcriptomic analysis was performed as implemented in the computational workflow for the detection of differentially expressed genes and pathways from RNA-seq data (54). Reads were aligned to the human GRCh37/hg19 (annotation NCBI_105.20190906) using HISAT2. Low-expression genes with at least one zero counts were filtered out and the remaining reads normalized with Relative Log Expression (RLE) method as implemented in DESeq2 R library. Differential gene expression between the control and treatment groups was estimated with DESeq2 Wald test (55). Sequencing files can be accessed on gene expression omnibus repository (GSE195855).

Gene Set Enrichment Analysis (GSEA) was performed on a pre-ranked GSEA list based on Log 2FC values of differentially expressed genes (DEGs: Log2FC > 1, p-value < 0.05), against Molecular Signatures Database (MsigDB v7.4) “Hallmark” and “Reactome” gene-sets. Weighted enrichment statistics were based on 1000 permutations. Significantly enriched gene-sets with FDR adjusted q-value <0.1 were selected for Enrichment map visualization as previously described (56, 57). Briefly, Enrichment files were inputted into the Enrichment Map app within the Cytoscape program for visualization (58). Parameters were set at default values (node cutoff FDR Q value 0.1; Jaccard Overlap combined coefficient cutoff 0.375, k-constant 0.5). Nodes were manually laid out and combined into common biological process for clarity using the AutoAnnotate app.

### Immunoblot

Treated cells were rinsed, lysed, subjected to SDS-PAGE and transferred to a polyvinylidene difluoride (PVDF) membrane as previously described (59). The following antibodies were used for immunoblotting: antirabbit and anti-mouse horseradish peroxidase-conjugated secondary antibodies (1:5000; Pierce); anti-GAPDH (1:2500; ab9485; Abcam); anti-human Hsp90 (1:1000, 610418, BD Biosciences); anti-pJAK2 (1:1000; 3774), anti-STAT3 (1:1000; 3743), anti-phosphoSTAT1 (1:1000; 9167), anti-IRF7 (1:1000; 4920), anti-IRF3 (1:1000; 11904) anti-IRF1 (1:1000; 8478), anti-pIRF7 (1:1000; 12390) all from Cell Signalling and anti-JAK2 (1:5000; 108596; Abcam). Blots were immersed in chemiluminescent substrate (SuperSignal West Pico Plus or Femto, Thermo Fisher Scientific), and signal was visualized using ChemiDoc MP imaging system (BIORAD).

### Interferon signalling

To study the modulation of the NF-κB and interferon regulatory factor (IRF) signalling pathways by the JAK inhibitors, 5 x 10^4^ A549-Dual™ hACE2-TMPRSS2 cells (*In vivo*Gen, Toulouse, France) were seeded per well in flat 96-well plates and incubated for 24 h with the compounds. The activities of the NF-κB and IRF signalling pathways were assessed by incubating the cell supernatants with QUANTI-Blue™ Solution and QUANTI-Luc™ respectively (*In vivo*Gen, Toulouse, France) according to manufacturer’s instructions. SEAP and luciferase were read in an EnSight™ multimode plate reader (PerkinElmer, Waltham, MA, USA).

### Tat-dependent viral expression of HIV-1 LTR promoter

HeLa TZM-bl cells harboring an integrated copy of HIV-1 LTR, controlling luciferase reporter gene expression was used for Tat-dependent viral transactivation assay. Cells were transfected or not with 10 – 50 ng of Tat-expressing plasmid (60) using Lipofectamine 3000 reagent (Invitrogen, Cat#L3000015) in a 24-well plate following manufacturer instructions. Cells were treated with compounds 24 h post transfection. HIV-1 Tat expression was then measured by a luciferase-based assay 20 h post drug treatment.

### Viral infection and antiviral activity

Activated CD4 + T cells from healthy donors were infected with the envelope-deficient HIV-1 NL4-3 clone (HIG) encoding internal ribosome entry site (IRES)-green fluorescent protein (GFP) (NL4-3-GFP) by spinoculation (1200×g, 1 h 30 min at 37°C). The anti-HIV activity of the different compounds was determined by the infection of cells in the presence of different concentrations of drugs, and 50% effective concentrations (EC50) were calculated, as previously described (61). Viral replication was measured 48 h later by quantification of GFP + expression by flow cytometry.

### Statistical analysis

Statistical significance for *in vitro* and *ex vivo* experiments was calculated using appropriate t test in Graphpad Prism (v9.3.0). All experiments were performed in at least three independent replicates and n values are provided in the figure legends. Plots were drawn using GraphPad Prism and R software.

## Data availability statement

The datasets presented in this study can be found in online repositories. The names of the repository/repositories and accession number(s) can be found below: gene expression omnibus (GSE195855).

## Ethics statement

The studies involving human participants were reviewed and approved by Ethics Committee of Hospital Germans Trias i Pujol (Ref. CEI PI-18–021). The patients/participants provided their written informed consent to participate in this study.

## Author contributions

Study conception and design: IE, BC, ER-M, and EB; Data collection: IE, EG-V, MP, BO-T, and LG-C; analysis and interpretation of results: MR-R, MM, JM-P, AG, RB, ER-M, and EB; Draft manuscript preparation: IE and EB. All authors reviewed the results and approved the final version of the manuscript.

## Funding

This work has been funded by Instituto de Salud Carlos III (ISCIII) through the project PI19/00194 (Co-funded by European Regional Development Fund/European Social Fund) “Investing in your future”) and in part by Grifols. IE is a research fellow from la Caixa (LCF/BQ/IN18/11660017) cofunded by the European Union’s Horizon 2020 research and innovation program under the Marie Sklodowska-Curie grant agreement No. 713673. EG-V is a research fellow from PERIS, supported by Ministry of Health of the Government of Catalonia (SLT017/20/000090). LG-C is a research fellow supported by the Secretariat of Universities and Research of the Government of Catalonia and European Social Fund (2019 FI-B00420). JM-P is supported by grants PID2019-109870RB-I00 from the Spanish Ministry of Science and Innovation and NIH/NIAID (RID-HIV: 1 UM1 AI164561-01). EF, RB, and EB are fellows from ISCIII (CM20/00027; CP19/00011 and CPII19/00012 respectively). We thank Foundation Dormeur for financial support for the acquisition of the QuantStudio-5 real time PCR system.

## Conflict of interest

The authors declare that the research was conducted in the absence of any commercial or financial relationships that could be construed as a potential conflict of interest.

## Publisher’s note

All claims expressed in this article are solely those of the authors and do not necessarily represent those of their affiliated organizations, or those of the publisher, the editors and the reviewers. Any product that may be evaluated in this article, or claim that may be made by its manufacturer, is not guaranteed or endorsed by the publisher.

## Supplementary material

Supplementary Figure 1 Determination of HIV-1 reactivation capacity in *in vitro* models of HIV-1 latency. HIV reactivation in *in vitro* lymphoid ACH2 model of latency **(A)** and in *in vitro* non-clonal myeloid latency model U-HIG **(B)**. HIV reactivation was determined by the intracellular expression of HIV-1 p24 antigen in ACH2 cells cultured for 48 h or by GFP+ expression in U-HIG cultured for 20 h as measured by flow cytometry in the presence of subtoxic concentrations of indicated compounds. HDCAi panobinostat (PNB) and vorinostat (VOR) were used as controls for HIV-1 reactivation. Bar plots represent relative HIV-1 reactivation and red dots represent cell viability of treatment conditions normalized to the untreated control (DMSO). Representative ELISPOT pictographs of HIV-1 CAp24 antigen capture in non-clonal models of HIV-1 latency J-HIG **(C)** and HL-HIG **(D)**. Pearson correlation plots of CAp24 antigen capture **(C, D)** versus relative HIV-1 reactivation capacity as measured by GFP detection (flow cytometry) of indicated compounds () in J-HIG **(E)** and HL-HIG **(F)** models of HIV latency. All statistical comparisons were performed with Student’s t tests. ∗p<0.05; ∗∗p<0.01; ∗∗∗p<0.001. Data are expressed as mean ± SD of at least three independent experiments.

Supplementary Figure 2 Gating strategy for the immunophenotypic characterization of PBMCs by flow cytometry. CD4 + T cell populations were gated on the live singlet CD3+CD4+ lymphocytes: T naïve T_N_ (CD45RA+CCR7+CD27+), T central memory T_CM_ (CD45RA-CCR7+CD27+), T transitional memory T_TM_ (CD45RA-CCR7-CD27+) and T effector memory T_EM_ (CD45RA-CCR7+CD27-). Immune activation markers HLA-DR+, CD25+ and CD69+ CD4+ lymphocytes were also gated on the live singlet CD3+CD4+ lymphocytes.

Supplementary Figure 3 Top 30 downregulated and upregulated genes following JAKi treatment in a model of HIV-1 latency (HL-HIG). Bar plots of top 30 differentially enriched genes (DEG) for each treatment condition (**A**, PMA; **B**, Fedratinib; **C**, Ruxolitinib and **D**, pacritinib) relative to the untreated control (ND), based on Log2 gene expression (Log2FC) and p < 0.05. Significantly down- or up-regulated DEG are highlighted in blue or red, respectively. **(E, F)** Confirmation of significantly top upregulated and downregulated genes in additional samples in myeloid HL60 and lymphoid Jurkat cell lines. Relative mRNA expression was measured by quantitative RT-PCR and normalized to GAPDH. All statistical comparisons were performed with Student’s t tests. *p<0.05; **p<0.01; ***p<0.001. Data are expressed as mean ± SD of at least three independent experiments.

Supplementary Figure 4 Reactome HIV budding, and maturation signalling is significantly enriched by fedratinib treatment in latently infected model. **(A)** Reactome Gene set enrichment map of significantly enriched pathways for fedratinib treatment condition. Reactome Gene set clusters are annotated, and nodes manually laid out for clarity (see also ). Single gene sets are highlighted and annotated in-text, underneath the cytoscape enrichment map. Node size represents number of genes, node colour represents significance (NES), and edge thickness represents number of shared genes. **(B)** Gene expression of leading-edge genes from the Reactome HIV Budding and Maturation signalling gene set. Fedratinib treatment condition was used as a reference set for leading-edge genes selection and ranking. Significantly down- or up-regulated gene sets **(A)** and genes **(B)** are highlighted in blue or red, respectively.

Supplementary Figure 5 Fedratinib potently blocks cytokine and NFκB signalling pathways *in vitro.* **(A, B)** IL8 and TNFα gene expression of JAKi-treated HL-60 (left panel) and Jurkat (right panel) cells. Relative mRNA expression was measured by quantitative RT-PCR and normalized to GAPDH. **(C)** Gene expression of leading-edge genes from the Hallmark TNFα *via* NFκB signalling gene set. Fedratinib treatment condition was used as a reference set for leading-edge genes selection and ranking. Significantly down- or up-regulated genes are highlighted in blue or red, respectively. **(D)** Induction of promoters for interferon stimulatory response element (ISRE) and NFκB by JAKi in A549-Dual™ hACE2-TMPRSS2 cells. All statistical comparisons were performed with Student’s t tests. **(E)** Luciferase activity of mock and HIV-1 Tat transfected HeLa TZM-bl cells. HeLa TZM-bl cells were transfected or not with Tat-expressing plasmid for 24h and HIV-1 Tat expression was measured by a luciferase-based assay. **(F)** Relative IRF7 mRNA expression of non-transfected TZM-bl cells treated with JAK2i for 20h. ∗p<0.05; ∗∗p<0.01; ∗∗∗p<0.001. Data are expressed as mean ± SD of at least three independent experiments.

Supplementary Table 1 Immunological and virological characteristics of study participants at the time of cell sample collection. * ABC, abacavir; COBI, cobicistat; DRV, darunavir; DTG, dolutegravir; FTC, emtricitabine; RAL, raltegravir; TAF, tenofovir alafenamide; 3TC, lamivudine.
