# Supplementary figures and images for: IRF7 expression correlates with HIV latency reversal upon specific blockade of immune activation
Source: Front Immunol. 2022 Sep 5;13:1001068. doi: 10.3389/fimmu.2022.1001068 (PMC9484258; doi:10.3389/fimmu.2022.1001068)

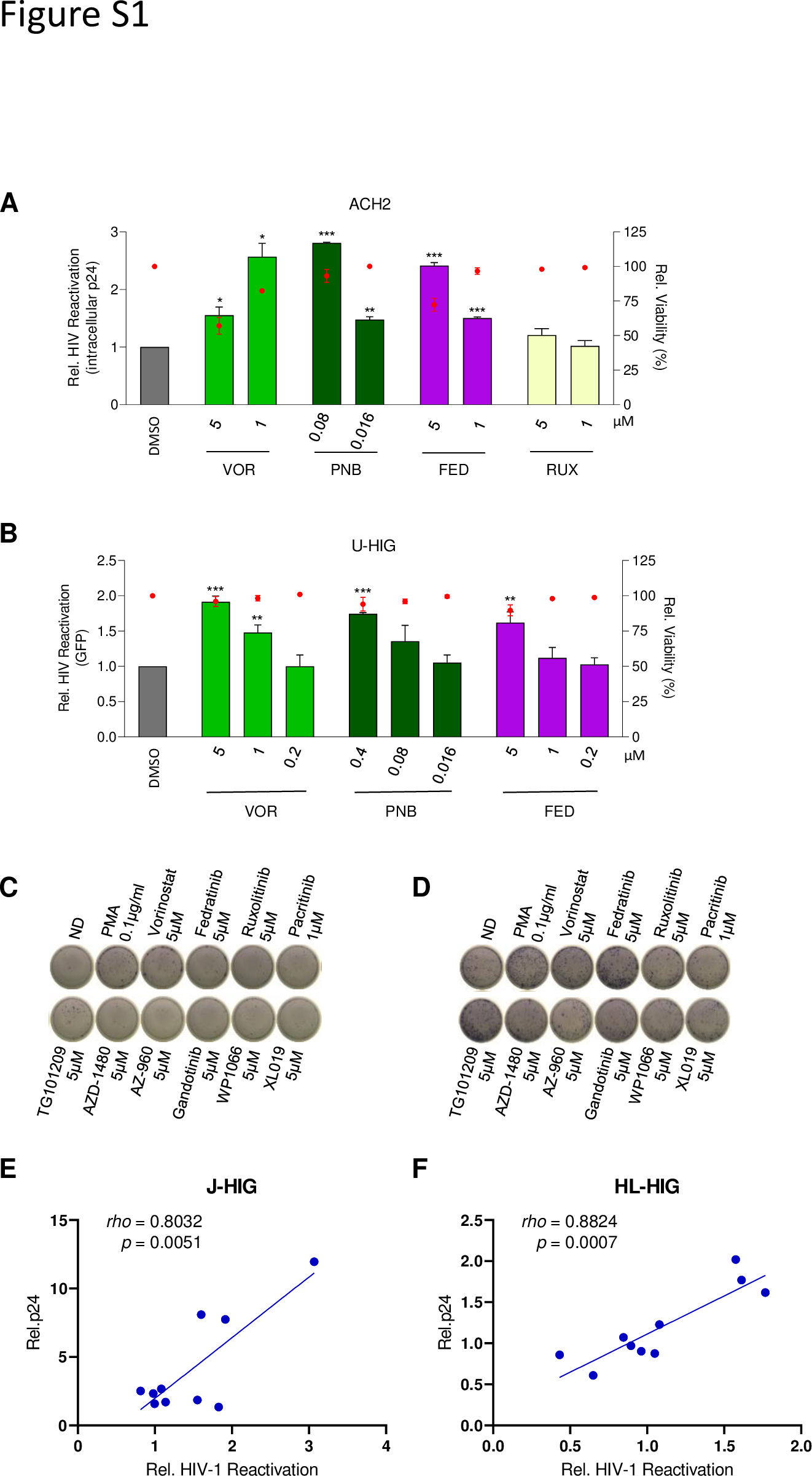

Supplement: Supplementary Figure 1 — Determination of HIV-1 reactivation capacity in in vitro models of HIV-1 latency. HIV reactivation in in vitro lymphoid ACH2 model of latency (A) and in in vitro non-clonal myeloid latency model U-HIG (B). HIV reactivation was determined by the intracellular expression of HIV-1 p24 antigen in ACH2 cells cultured for 48 h or by GFP+ expression in U-HIG cultured for 20 h as measured by flow cytometry in the presence of subtoxic concentrations of indicated compounds. HDCAi panobinostat (PNB) and vorinostat (VOR) were used as controls for HIV-1 reactivation. Bar plots represent relative HIV-1 reactivation and red dots represent cell viability of treatment conditions normalized to the untreated control (DMSO). Representative ELISPOT pictographs of HIV-1 CAp24 antigen capture in non-clonal models of HIV-1 latency J-HIG (C) and HL-HIG (D). Pearson correlation plots of CAp24 antigen capture (C, D) versus relative HIV-1 reactivation capacity as measured by GFP detection (flow cytometry) of indicated compounds () in J-HIG (E) and HL-HIG (F) models of HIV latency. All statistical comparisons were performed with Student’s t tests. ∗p<0.05; ∗∗p<0.01; ∗∗∗p<0.001. Data are expressed as mean ± SD of at least three independent experiments. [file Image_1.tif]

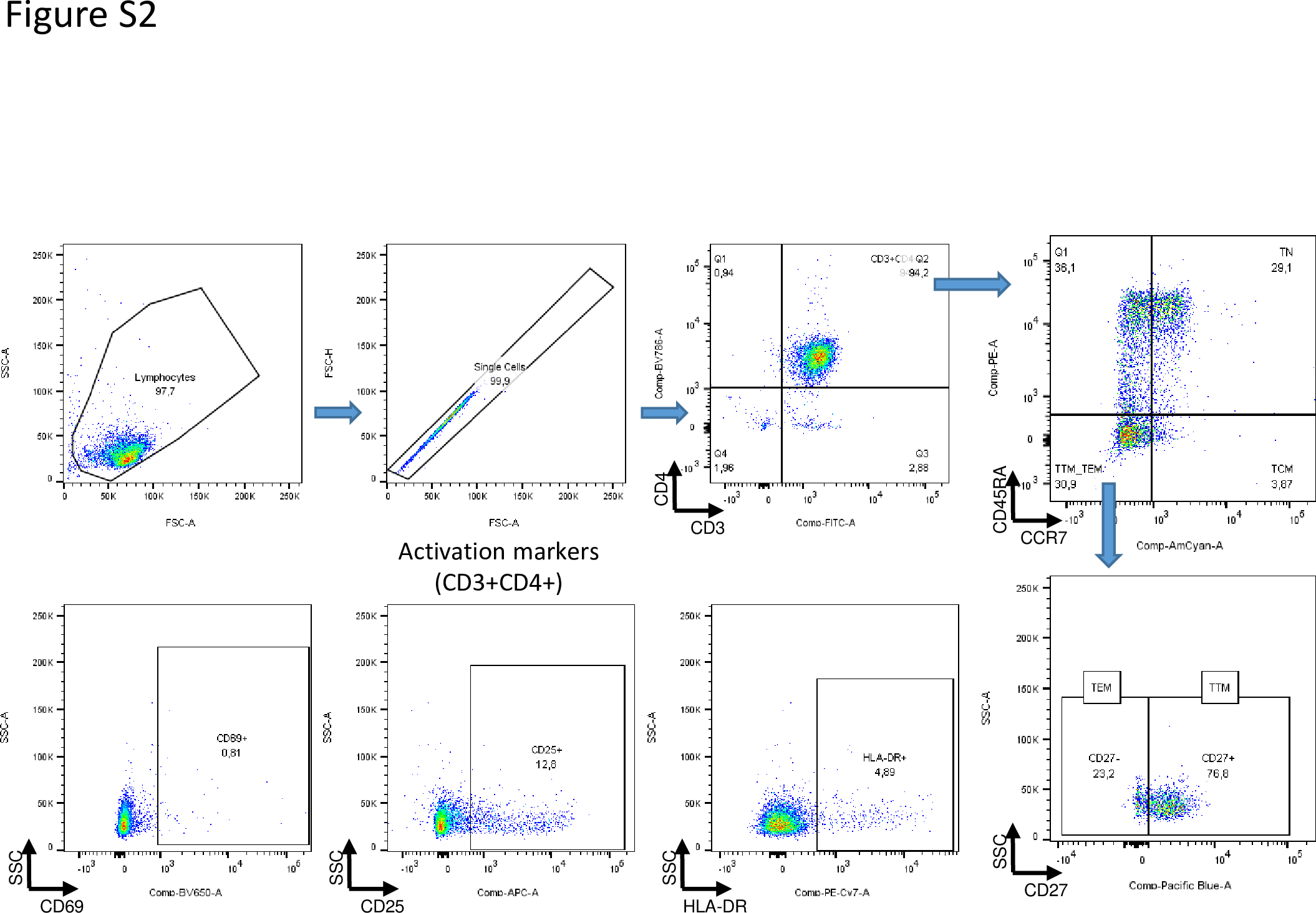

Supplement: Supplementary Figure 2 — Gating strategy for the immunophenotypic characterization of PBMCs by flow cytometry. CD4 + T cell populations were gated on the live singlet CD3+CD4+ lymphocytes: T naïve TN (CD45RA+CCR7+CD27+), T central memory TCM (CD45RA-CCR7+CD27+), T transitional memory TTM (CD45RA-CCR7-CD27+) and T effector memory TEM (CD45RA-CCR7+CD27-). Immune activation markers HLA-DR+, CD25+ and CD69+ CD4+ lymphocytes were also gated on the live singlet CD3+CD4+ lymphocytes. [file Image_2.tif]

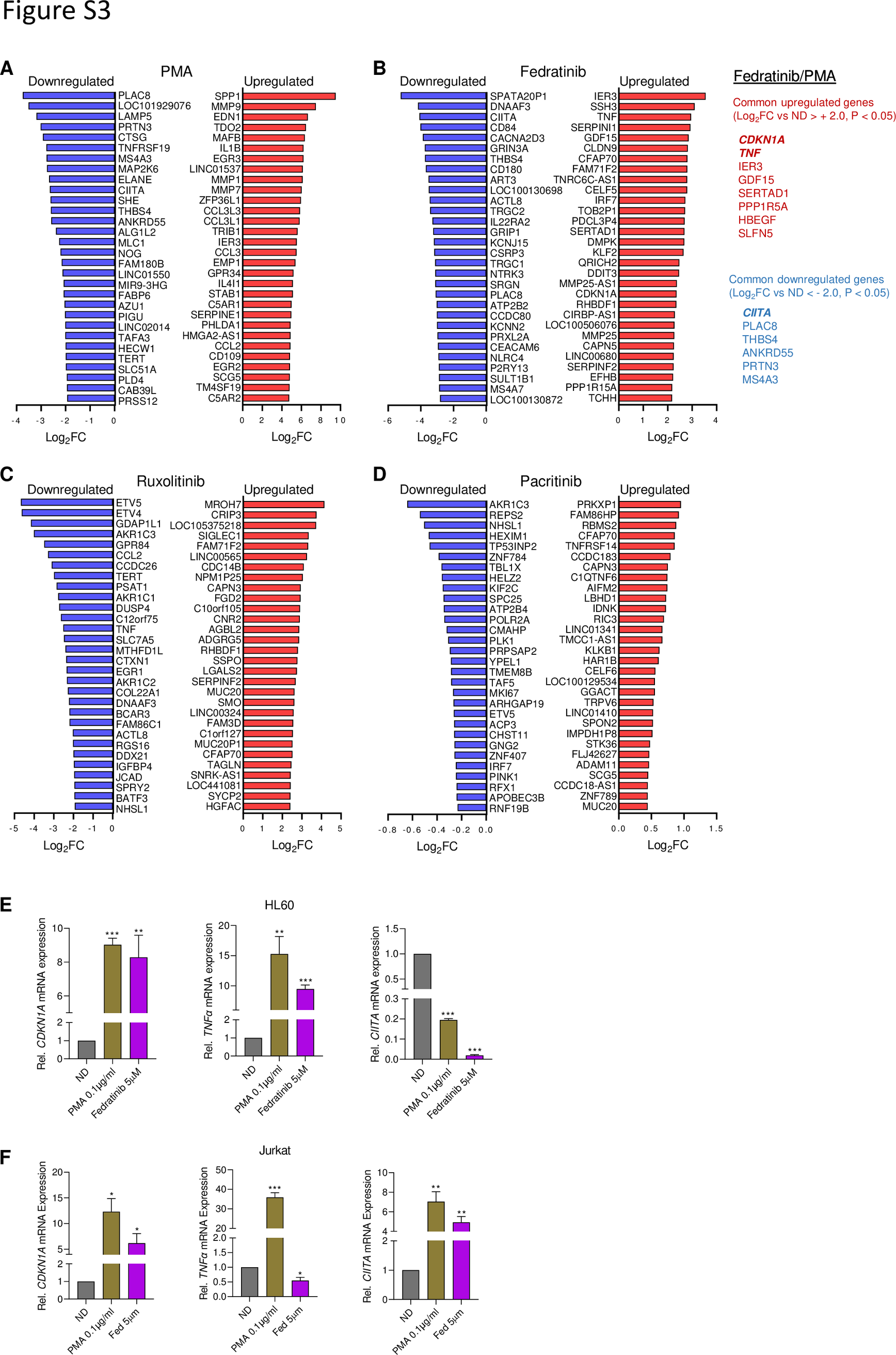

Supplement: Supplementary Figure 3 — Top 30 downregulated and upregulated genes following JAKi treatment in a model of HIV-1 latency (HL-HIG). Bar plots of top 30 differentially enriched genes (DEG) for each treatment condition (A, PMA; B, Fedratinib; C, Ruxolitinib and D, pacritinib) relative to the untreated control (ND), based on Log2 gene expression (Log2FC) and p < 0.05. Significantly down- or up-regulated DEG are highlighted in blue or red, respectively. (E, F) Confirmation of significantly top upregulated and downregulated genes in additional samples in myeloid HL60 and lymphoid Jurkat cell lines. Relative mRNA expression was measured by quantitative RT-PCR and normalized to GAPDH. All statistical comparisons were performed with Student’s t tests. *p<0.05; **p<0.01; ***p<0.001. Data are expressed as mean ± SD of at least three independent experiments. [file Image_3.tif]

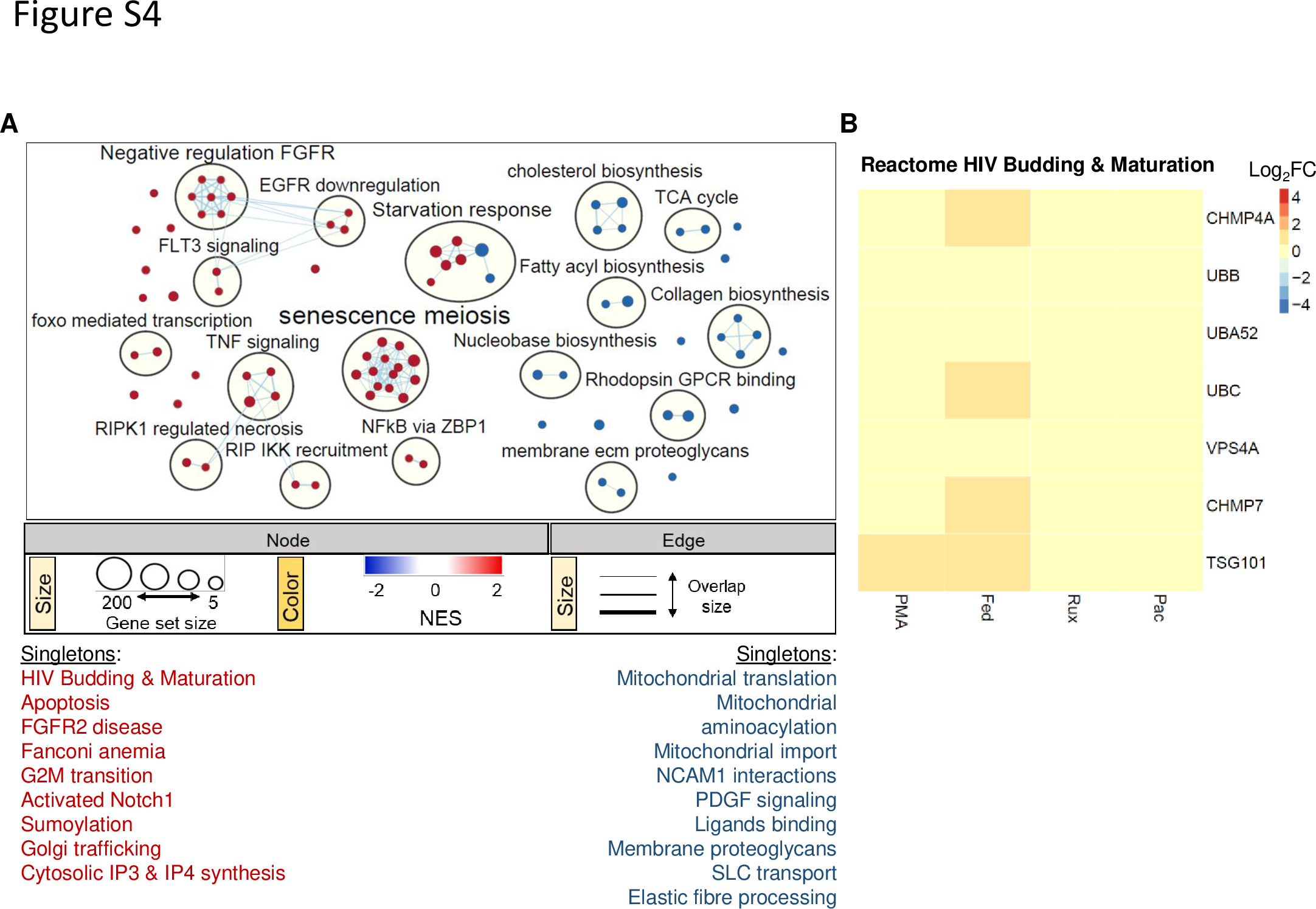

Supplement: Supplementary Figure 4 — Reactome HIV budding, and maturation signalling is significantly enriched by fedratinib treatment in latently infected model. (A) Reactome Gene set enrichment map of significantly enriched pathways for fedratinib treatment condition. Reactome Gene set clusters are annotated, and nodes manually laid out for clarity (see also ). Single gene sets are highlighted and annotated in-text, underneath the cytoscape enrichment map. Node size represents number of genes, node colour represents significance (NES), and edge thickness represents number of shared genes. (B) Gene expression of leading-edge genes from the Reactome HIV Budding and Maturation signalling gene set. Fedratinib treatment condition was used as a reference set for leading-edge genes selection and ranking. Significantly down- or up-regulated gene sets (A) and genes (B) are highlighted in blue or red, respectively. [file Image_4.tif]

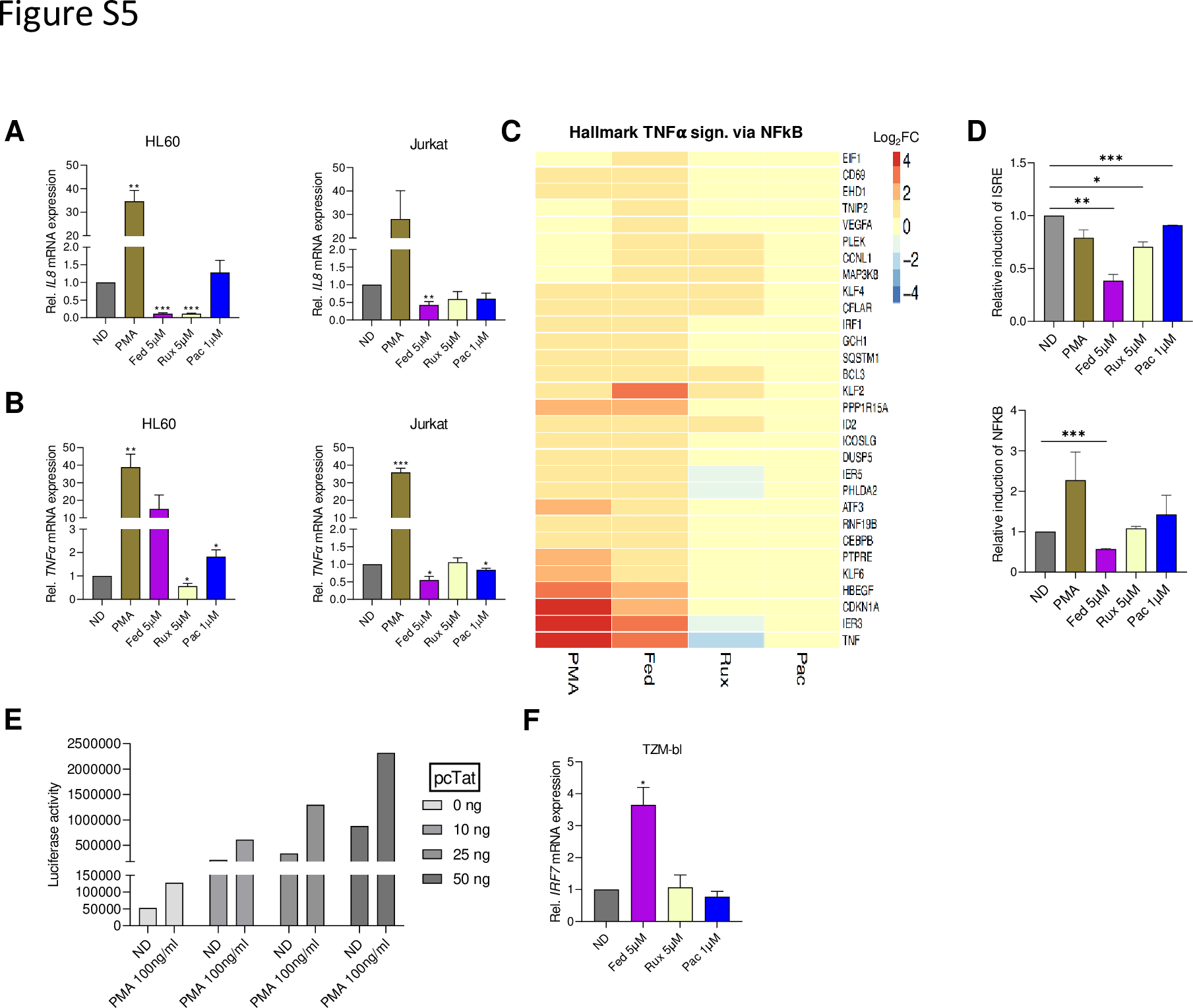

Supplement: Supplementary Figure 5 — Fedratinib potently blocks cytokine and NFκB signalling pathways in vitro. (A, B) IL8 and TNFα gene expression of JAKi-treated HL-60 (left panel) and Jurkat (right panel) cells. Relative mRNA expression was measured by quantitative RT-PCR and normalized to GAPDH. (C) Gene expression of leading-edge genes from the Hallmark TNFα via NFκB signalling gene set. Fedratinib treatment condition was used as a reference set for leading-edge genes selection and ranking. Significantly down- or up-regulated genes are highlighted in blue or red, respectively. (D) Induction of promoters for interferon stimulatory response element (ISRE) and NFκB by JAKi in A549-Dual™ hACE2-TMPRSS2 cells. All statistical comparisons were performed with Student’s t tests. (E) Luciferase activity of mock and HIV-1 Tat transfected HeLa TZM-bl cells. HeLa TZM-bl cells were transfected or not with Tat-expressing plasmid for 24h and HIV-1 Tat expression was measured by a luciferase-based assay. (F) Relative IRF7 mRNA expression of non-transfected TZM-bl cells treated with JAK2i for 20h. ∗p<0.05; ∗∗p<0.01; ∗∗∗p<0.001. Data are expressed as mean ± SD of at least three independent experiments. [file Image_5.tif]
