# Supplementary material for: IRF7 expression correlates with HIV latency reversal upon specific blockade of immune activation
Source: Front Immunol. 2022 Sep 5;13:1001068. doi: 10.3389/fimmu.2022.1001068 (PMC9484258; doi:10.3389/fimmu.2022.1001068)
Supplement: Supplementary Table 1 — Immunological and virological characteristics of study participants at the time of cell sample collection. * ABC, abacavir; COBI, cobicistat; DRV, darunavir; DTG, dolutegravir; FTC, emtricitabine; RAL, raltegravir; TAF, tenofovir alafenamide; 3TC, lamivudine. [file Table_1.docx]

**Table S1. Immunological and virological characteristics of study participants at the time of cell sample collection**

| ID | Age | Sex | Ethnicity | Estimated min. length of HIV infection  (years) | Estimated min. length of viral suppression (years) | CD4 Nadir (cells/µl) | CD4 count (cells/µl) | Viral Load  (copies/ml) | ART Regimen* |
| --- | --- | --- | --- | --- | --- | --- | --- | --- | --- |
| p1 | 56 | M | Caucasian | 13 | 10 | 535 | 1075 | <40 | RAL/FTC/TAF |
| p2 | 44 | M | Caucasian | 23 | 11 | 455 | 908 | <40 | DTG/ABC/3TC |
| p3 | 38 | M | Caucasian | 4 | 4 | 460 | 932 | <40 | DRV/COBI |
| p4 | 41 | M | Caucasian | 8 | 6 | 413 | 1002 | <40 | DTG/3TC |

* ABC, abacavir; COBI, cobicistat; DRV, darunavir; DTG, dolutegravir; FTC, emtricitabine; RAL, raltegravir; TAF, tenofovir alafenamide; 3TC, lamivudine
